# Fast Mode Decision in the HEVC Video Coding Standard by Exploiting Region with Dominated Motion and Saliency Features

**DOI:** 10.1371/journal.pone.0150673

**Published:** 2016-03-10

**Authors:** Pallab Kanti Podder, Manoranjan Paul, Manzur Murshed

**Affiliations:** 1 School of Computing & Mathematics, Charles Sturt University, Bathurst, NSW, Australia; 2 School of Information Technology, Federation University, Churchill, VIC, Australia; Beijing University of Technology, CHINA

## Abstract

The emerging *High Efficiency Video Coding* (HEVC) standard introduces a number of innovative and powerful coding tools to acquire better compression efficiency compared to its predecessor H.264. The encoding time complexities have also increased multiple times that is not suitable for realtime video coding applications. To address this limitation, this paper employs a novel coding strategy to reduce the time complexity in HEVC encoder by efficient selection of appropriate block-partitioning modes based on *human visual features* (HVF). The HVF in the proposed technique comprise with human visual attention modelling-based saliency feature and phase correlation-based motion features. The features are innovatively combined through a fusion process by developing a content-based adaptive weighted cost function to determine the *region with dominated motion/saliency* (RDMS)- based binary pattern for the current block. The generated binary pattern is then compared with a codebook of predefined binary pattern templates aligned to the HEVC recommended block-paritioning to estimate a subset of inter-prediction modes. Without exhaustive exploration of all modes available in the HEVC standard, only the selected subset of modes are motion estimated and motion compensated for a particular coding unit. The experimental evaluation reveals that the proposed technique notably down-scales the average computational time of the latest HEVC reference encoder by 34% while providing similar *rate-distortion* (RD) performance for a wide range of video sequences.

## Introduction

Developing a number of powerful coding tools, the latest HEVC [1] video coding standard has provided similar perceptual image quality compared to its predecessor H.264 [2] at approximately 50% bit-rate reduction due to efficient transmission and storage of high volume video data [3]. HEVC achieves this improved performance at the cost of more than 4 times algorithmic complexity due to the extended number of levels and complex *coding unit* (CU) partitioning scheme compared to its predecessor H.264 in a particular implementation [4,5]. For this reason, any electronic devices with limited processing capacity could not fully exploit HEVC encoding and decoding features. This motivated us to reduce the computational time of HEVC encoder by appropriate selection of inter-prediction modes. For this to happen, only the RDMS in a video is taken into account that comprise with visually attentive area based saliency feature and phase correlation based motion features.

Through the mode selection process, many researchers have contributed in different ways to reduce computational complexity of HEVC reference test model in terms of both inter and intra coding. To alleviate the computational complexity of HEVC encoder, Shen *et al*. [6] propose a fast CU size decision and intra-mode decision approach by utilizing different CU depth ranges. They adopt the technique to reduce the complexity of computation by avoiding a number of prediction modes not often used in the parent CUs in upper depth levels or nearby CUs. Experimentally they can save on average 21% computational complexity of HEVC encoder with negligible loss of coding efficiency. Recently, the authors in [7] propose an effective CU size decision algorithm for HEVC intra prediction. The algorithm is developed in two aspects: (i) the early CU size decision using adaptive thresholds by analysing texture homogeneity, and (ii) for the large size CUs, adopting the strategy of avoiding the in-depth intra prediction. The simulation results demonstrate that their technique obtain a complexity reduction up to 67% although it incurs with the loss of 0.06 dB *peak signal-to-noise ratio* (PSNR) or 1.08% bit-rate increment. A fast *prediction unit* (PU) skip and split termination algorithm for HEVC intra-prediction is proposed by Lim *et al* [8]. The early skip, PU skip and PU split algorithms perform- immediate skipping of RD cost computation for large PUs, skipping of full RD cost computation and terminating further PU splitting using the RD cost respectively. Using their algorithms, the experimental results reveal 44.05% time savings on average compared to *HEVC reference test model* (HM) with similar RD performance.

Several authors have proposed heuristics for simplifying the partitioning decision for CUs, PUs, and *transform units* (TUs), aiming at decreasing the computational complexity of HEVC encoder through inter-mode selection [9–13]. Recently, Shen *et al*. [14] introduce a TU size decision based early termination algorithm for HEVC reference encoders by using the Bayesian decision theory and finding correlation between residual coefficients and transform size variance to decrease the number of candidate transform sizes. The experimental results confirm that their proposed algorithm is capable of saving 30–46% of processing complexity with some losses in coding efficiency. For faster CU encoding, Ahn *et al*. [15] utilizes the spatiotemporal encoding parmetres of HEVC encoders. Actually, they make use of sample-adaptive-offset parameters as the spatial encoding parameter, while, the motion vectors, TU size, and coded block flag information as the temporal encoding parameters to estimate texture and temporal complexity of a CU. Experimental results show 46% encoding time reduction on average with the bit-rate increment of 1.2%. The authors in [16] propose an early skip mode decision technique to reduce encoding complexity of HEVC encoder. The skip mode for a prediction unit is determined by calculating the RD cost of 2N×2N merge mode and the threshold for early decision is determined from the video data. Once a mode is decided as a merge mode do not undergo further partitioning. Experimental results shows that proposed method alleviate on average 28% encoding complexity of HEVC encoder with almost no coding loss. The technique presented in [17] by Shen *et al*. jointly utilizes **t**he inter-level correlation of quadtree structure and the spatiotemporal correlation to fasten the intermode decision for HEVC. From the existing correlations of prediction mode, motion vector, and RD cost of coding depth levels, they analyse the prediction mode distribution and coding correlation of adjacent CUs. They eventually propose three adaptive intermode decision techniques that results in 49%-52% computational complexity reduction with negligible loss of coding efficiency.

Hou *et al*. [18] recommend a RD cost based threshold to explore modes only in the higher level that results in 30% time savings with 0.5% quality loss. Vanne *et al*. [19] propose an efficient inter-mode decision scheme by finding the candidate modes of symmetric and asymmetric motion partition. The tested results reveals the reduction of HEVC encoder complexity by 31%-51% at the cost of 0.2%-1.3% bit-rate increment. Pan *et al*. [20] introduce an early MERGE mode decision algorithm to reduce computational complexity of HEVC encoder. Based on all zero block and motion information, they first apply MERGE mode for the root CUs then for the children CUs by mode selection correlation. They achieve 35% time savings with the bit-rate increment of 0.32%, and quality loss of 0.11 dB *peak signal to noise ratio* (PSNR). Shen *et al*. [21] introduce checking criteria based early termination method which selects 36% and 14% of modes at depth level ‘0’ and ‘3’ respectively. This process incurs with quality loss especially for sequences containing a large area with high motion activities although their algorithm saves around 30% of computing time.

In addition to the above mentioned mode selection algorithms based on HEVC video coding standard, other fast mode selection algorithms based on H.264 video coding standard are also available in the literature [22–24]. Paul *et al*. [25] extract *energy concentration ratio* (ECR) from phase correlation and employ it for modes selection process to reduce encoding time in H.264 standard. The approach used in [25] would not be straightforward applied in HEVC to select the direct mode or a subset of inter-modes due to the three times extended number of modes, double/quadruple size of CUs, and complex (i.e., symmetric/asymmetric) CU partitioning patterns compared to H.264. Moreover, only the ECR based mode selection would not provide expected compression results in HEVC as it indicates only the residual error between current block and the motion-compensated reference block. It also unnecessarily uses smaller block-partitions while a block does not have any translational motion or provides high accurate predicted motion.

For more accurate decision on *motion estimation* (ME) and *motion compensation* (MC) modes, in this paper, we extract three motion features including ECR, phase correlation peak and motion vector information from phase correlation to overcome the constraints in [25]. Since human visual system is also sensitive for different colours, brightness, and contrast in static areas, therefore, other than motion, we also capture visual attentive areas by applying the saliency modelling on the current image blocks. Thus, the RDMS which is sensitive to HVS for quality assessment is comprised with the combination of three motion features and one saliency feature in the proposed technique. We incorporate them by developing an adaptive weighted cost function to actuate RDMS based binary pattern for the current block. The resultant binary pattern is then compared against a codebook of predefined binary pattern templates aligned to the HEVC recommended block-partitioning to estimate a subset of inter-modes. From the selected subset of inter-modes, the final mode is determined based on their lowest value of the Lagrangian cost function. The proposed method not only reduces the computational time by appropriate selection of RDMS based ME and MC modes but also demonstrates the similar RD performance.

The major contributions of this paper are summarized as follows: (i) We introduce three motion features of phase correlation and exploit them for fast mode selection process in HEVC standard, (ii) We include the saliency in our algorithm and effectively use this feature for RDMS categorization, (iii) For the RDMS determination, we adaptively design the binary pattern templates based on the CU partitioning patterns in HEVC, and (iv) We develop a content-based adaptive weighted cost function through features fusion and innovatively derive weights for each feature adaptively.

The rest of the paper is organized as follows: Section 2 articulates working mechanism of recent HEVC, Section 3 describes the key steps of the proposed coding technique; Section 4 evaluates the experimental results and discussions in detail, while Section 5 concludes the paper.

## Recent HEVC Analysis

Compared to the state-of-the-art H.264 standard, HEVC introduces inventive approaches including the CU size extension from 16×16 up to 64×64-pixels, variable size PU and TU, and the symmetric/asymmetric block partitioning phenomenon. To select a particular motion prediction mode, HM checks the *Lagrangian cost function* (LCF) [26] exhaustively using all modes in each coding depth level (level 64×64, 32×32, 16×16 and 8×8 are denoted as depth level 0, 1, 2, 3 respectively). The LCF, *J*_*n*_ for the *n*th mode selection is defined by:

$$J_{n}=D_{n}+\lambda \times R_{n}$$ (1)

where *λ* is the Lagrangian multiplier, *D* is the distortion, and *R* is the resultant bit, which are determined by modes for a CU. In order to select the best partitioning mode in a coding depth level, the HM checks minimum 8 (i.e., 64×64, 64×48, 48×64, 64×32, 32×64, 16×64, 64×16, and 32×32), and maximum 24 inter-prediction modes (i.e., similar partitioning with smaller blocks from 32×32 to 8×8) with lowest LCF. This process is extremely time consuming due to the exploration of all modes in one or more coding depth levels. Unlike *HEVC test model* (HM12.1) [27], in the proposed technique, the selected CUs with RDMS are motion estimated and motion compensated with modes in the higher depth levels, on the other hand, the CUs without RDMS are motion estimated and motion compensated with modes in lower depth levels. Thus, we can avoid exhaustive exploration of all modes in each coding depth level. This results in computational time reduction.

**Fig 1:**
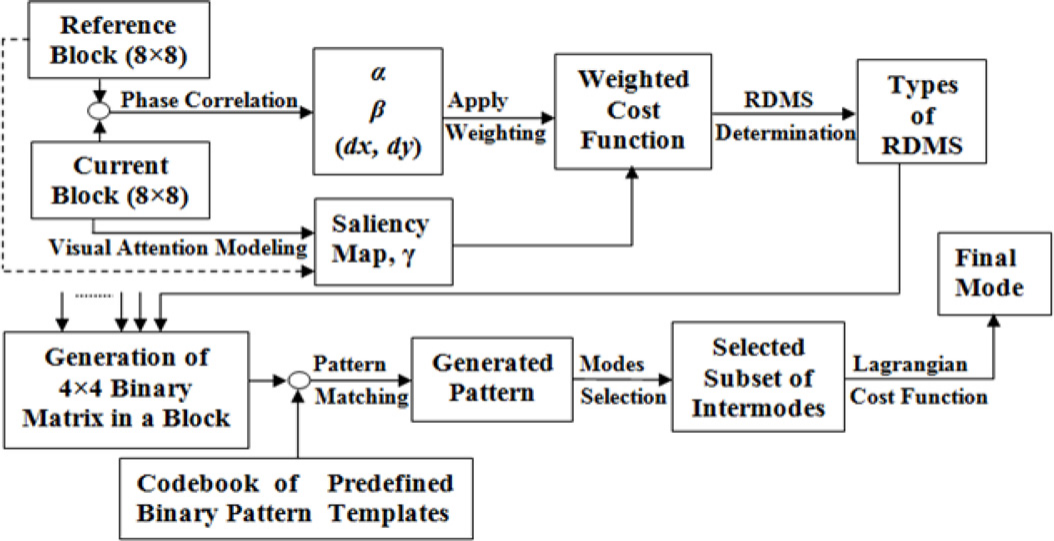
Process diagram of the proposed mode selection technique.

In terms of HM based mode selection, we noticed from Eq (1) that the best mode which is selected for a given value of Lagrangian Multiplier (i.e., *λ*), would be different for other values of that of the multiplier. Thus, the different multiplier values may select different best modes in a coding depth level for a given *quantization parameter* (QP). Moreover, only the LCF-based mode decision could not provide the best RD performance at different operational coding points because of complex CU partitioning patterns, block-partitioning and transformation headers, coding length of motion vectors, diversified video contents, and other advanced parameter settings in the HEVC video coding standard. Therefore instead of merely depending on the existing LCF, in the first phase, the proposed technique concentrates on RDMS criteria for a subset of intermode selection which is independent from the existing LCF. These number of consecutive pre-processing stages (shown in Fig 1) make the mode decision process more appropriate and less time consuming.

## Proposed Technique

In the proposed encoding method, we use 64×64 CU size and encode all the inter-modes at level 0 exhaustively using LCF similar to the HM. If 32×32 mode is selected then we use phase correlation to reduce the computational time from that level to higher levels, i.e., level 1 to 3. As the probability of selecting a 64×64 partition size for the video sequences with mid to lower range resolution is below 10%, we do not apply the proposed phase correlation strategy for level 0. The phase-correlation provides us the relative displacement information between current block and the reference block by *Fast Fourier Transformation* (FFT). In order to calculate an approximation of the corresponding motion between two blocks from the current and reference images, we apply the phase correlation technique. In this paper, we exploit phase correlation based three motion features including: (i) energy concentration ratio (*α*), (ii) predicted motion vector (*dx*,*dy*), and (iii) phase correlation peak (*β*) that focus on three different aspects of motions. Visual attentive portions from the video contents are captured by saliency feature (*γ*) of *graph based visual saliency* (GBVS) modeling. This GBVS is applied on the current image blocks so that it could calture the best salient salient points based on human visual perception and attention. Then we develop a cost function through a fusion process based on the RDMS using weighted average of the saliency values and the normalized motion features to determine a unified RDMS feature for the current block. Finally we configure the binary RDMS pattern from the unified RDMS features by applying predefined threshold. The best-matched pattern template is selected using a similarity metric where each of the templates corresponds to a sub-set of inter-prediction modes. From the selected subset, the final mode is determined based on the lowest value of the LCF. The entire process is shown as a process diagram in Fig 1.

### Motion Features Extraction

We calculate the phase correlation by applying the FFT and then *inverse* FFT (IFFT) of the current and reference blocks and finally applying the FFTSHIFT function as follows:

$$\Omega =\Gamma \;|\;\Lambda \;(e^{j(∠F_{r}\text{-}∠F_{c})})\;|$$ (2)

where *F*_*c*_ and *F*_*r*_ are the Fast Fourier transformed blocks of the current *C* and reference *R* blocks respectively, Г is the FFTSHIFT, Ʌ denote the IFFT and ∠ is the phase of the corresponding transformed block. Note that Ω is a two dimensional matrix. We evaluate the phase correlation peak (*β*) from the position of (dx + μ/2 + 1, dy + μ/2 + 1) as follows:

β=Ω(dx+μ/2+1,dy+μ/2+1) (3)

where the blocksize denoted by *μ* is 8 since we exploit 8×8-pixel block for phase correlation. Then we compute the predicted motion vector (*dx*, *dy*) by subtracting *μ*-1 from the (*x*, *y*) position of Ω where we find the maximum value of Ω. In the matched block generation process, we use the phase of the current block and magnitude of the motion-compensated block in the reference frame and finally calculate the matched reference block (Ѱ) for the current block by:

$$\Psi =\left|\Lambda (|F_{r}|e^{j(∠F_{c})})\right|$$ (4)

Now the displacement error (§) is enumerated by:

§=C–Ψ (5)

We then apply the *discrete cosine transform* (DCT) to error § and calculate the ECR (i.e., α) as the ratio of low frequency component and the total energy of the error block (i.e., ratio from the top-left triangle energy with respect to the whole area energy) by:

$$\alpha =(D_{EL}/D_{ET})$$ (6)

where *D*_*EL*_ and *D*_*ET*_ represent the top-left triangle energy and the whole area energy of a particular block. Note that the two sides of the top-left triangle are three-fourth of the blocksize i.e., 6 in our implementation.

### Saliency Feature Extraction

Salient areas may be considered in an image with high motion, high resolution or coloured region. As an example, the authors in [28] introduce a saliency detection technique in the videos by exploiting superpixel-based spatiotemporal saliency model. They also capture motion and colour histograms as the important features at superpixel level. Thus, saliency map gives us the prominent and interesting regions in an image based on human visual perception and attention (visual attention based rate control algorithm is detailed in [29]). However, the leading models of visual saliency may consist of (i) extracting feature vectors (ii) forming an activation map using the feature vectors and (iii) normalizing the activation map [30,31]. We exploit the saliency map as a tool in this paper (based on [30,32]) and incorporate into our coding architecture. The exploited GBVS technique gives us the variance map of RDMS based human visual features for an 8×8-pixel block consisting of the values ranging from 0 to 1. For a given 8×8 block, we extract a salience feature, *γ*, by averaging all values. In the proposed scheme, we incorporate this map into the cost function to determine the visually attentive areas with/without motion. Therefore, this feature would effectively capture the visually sensitive regions and our focus is to encode the RDMS based salient portions with more bits not only to achieve better compression efficiency but also for coding performance improvement.

### RDMS Categorization through Feature Fusion

After evaluating the phase correlation extracted motion features (i.e., *α*, *β* and (*dx*, *dy*) and saliency extracted variance map (i.e., γ), we finally determine a cost function. The development of the content-based adaptive weighted cost function for a block is carried out through a feature fusion process. The equation for the cost function is given by:

$$¥(i,j)=\omega_{1}\alpha (i,j)+\omega_{2}(1-\beta )+\omega_{3}\left(\frac{\left|dx\right|}{\delta }+\frac{\left|dy\right|}{\delta }\right)+\omega_{4}(\gamma )$$ (7)

where *δ* denote the block size, and *ω*_1_ to *ω*_4_ are the weights with $\sum_{i=1}^{4}\omega_{i}=1.$ We innovatively derive weights for each feature adaptively and do not consider all possible weight-combinations in this experiment. We only consider 0.50, 0.25, 0.125, and 0.125 weights based on the relative texture deviation of the current block against that of the whole frame. To calculate the deviation we apply *Standard Deviation* (STD) both on the current block and the current frame and use those weights for four attributes. First, we sort four features based on their values and if the value of the STD of the block is smaller than the value of the current frame then the highest weight (i.e., 0.50) is applied to the feature 1 (i.e., sorted) and the lowest weight (i.e., 0.125) is applied to the feature 4 (according to the sorted list); otherwise, inverse weighted order is applied. If the resultant value of the cost function (i.e., ¥) is greater than a predefined threshold the block is tagged by ‘1’ otherwise tagged by ‘0’ where ‘1’ and ‘0’ corresponds to RDMS and non- RDMS respectively.

The rationality of the proposed weight selection strategy is that if the current block has higher texture variation compared to the current frame, the current block should be encoded with more bits compared to the rest of the blocks to achieve similar/improved RD performance. To ensure spending more bits we need to categorize the block as RDMS block which is done by our threshold selection strategy. Other weight selection approach might work better, however, the experimental results show that the proposed technique provides similar RD performance.

**Fig 2:**
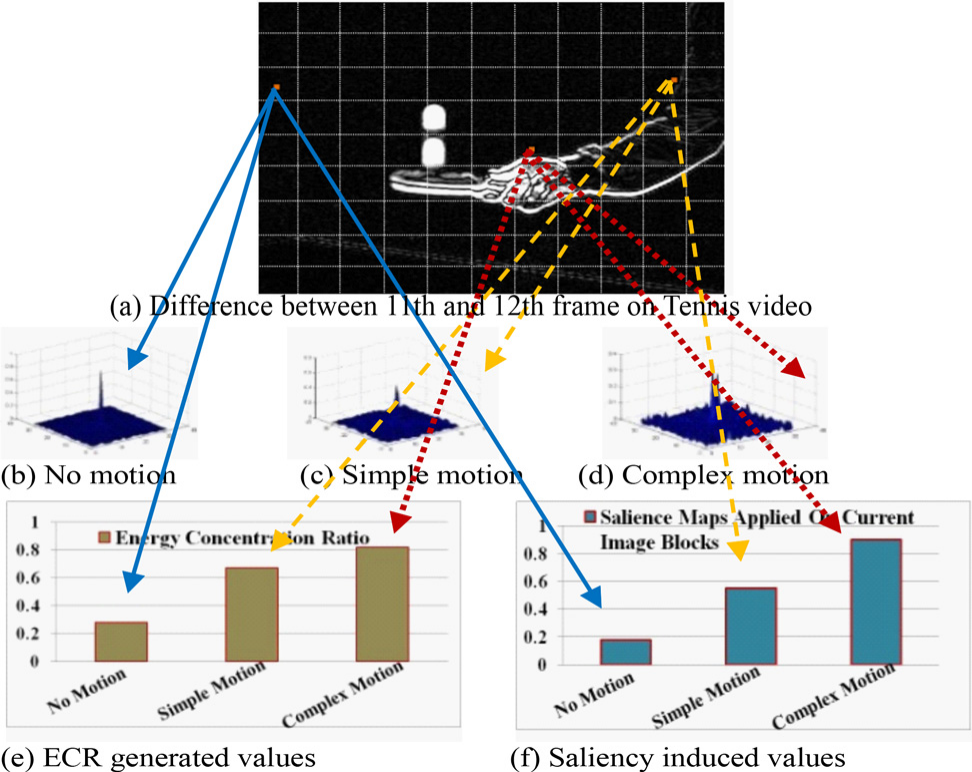
Illustration of motion features and saliency feature generated at different blocks of 12^th^ frame on *Tennis* video; (b~d) are the phase shifted plots for no motion (0.4), simple motion (0.7) and complex motion (0.8); (e-f) corresponds to the respective values generated by ECR and saliency feature for blocks at positions (3, 1), (3, 10) and (5, 7) respectively. For clear visualization, we use 32×32 block size.

**Fig 3:**
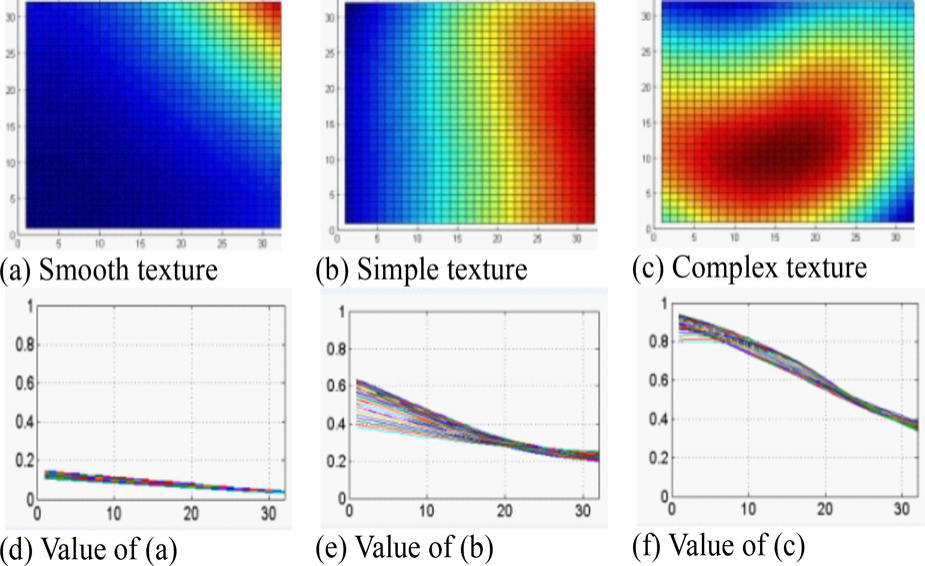
Salience feature after applying GBVS on three different image blocks (GBVS modelling applied on the current image blocks) with different motions and texture of a frame identified in Fig 2; note that for clear visualization we use 32×32-pixel block.

**Fig 2** demonstrates the relationship of the quantitative motion and salience features with the human visual features. **Fig 2B–2D**) shows the categories of motion-peak (*β*) and their corresponding values provided by ECR (in **Fig 2(E))** and saliency feature (in **Fig 2(F)**) for *Tennis* video. It is obvious from the figure that for *α*, and *γ*, the values of complex motion are the highest, while, for *β*, the complex motion has multiple peaks and it’s value is the lowest. The applied GBVS technique produces the resultant cost function based actual salience maps. These maps are generated between 11^th^ and 12^th^ frame on *Tennis* video for CU at positions (3, 1), (3, 10) and (5, 7) respectively with its texture deviation as illustrated in **Fig 3**. From the figure and the experimental results, we can easily observe that the features *α*, (*dx*, *dy*), and *γ* have positive correlation and *β* has inverse correlation to indicate human visual features. We level the complex texture and smooth texture areas by reddish and bluish colour respectively while any other colour corresponds to simple texture areas in **Fig 3**.

**Fig 4:**
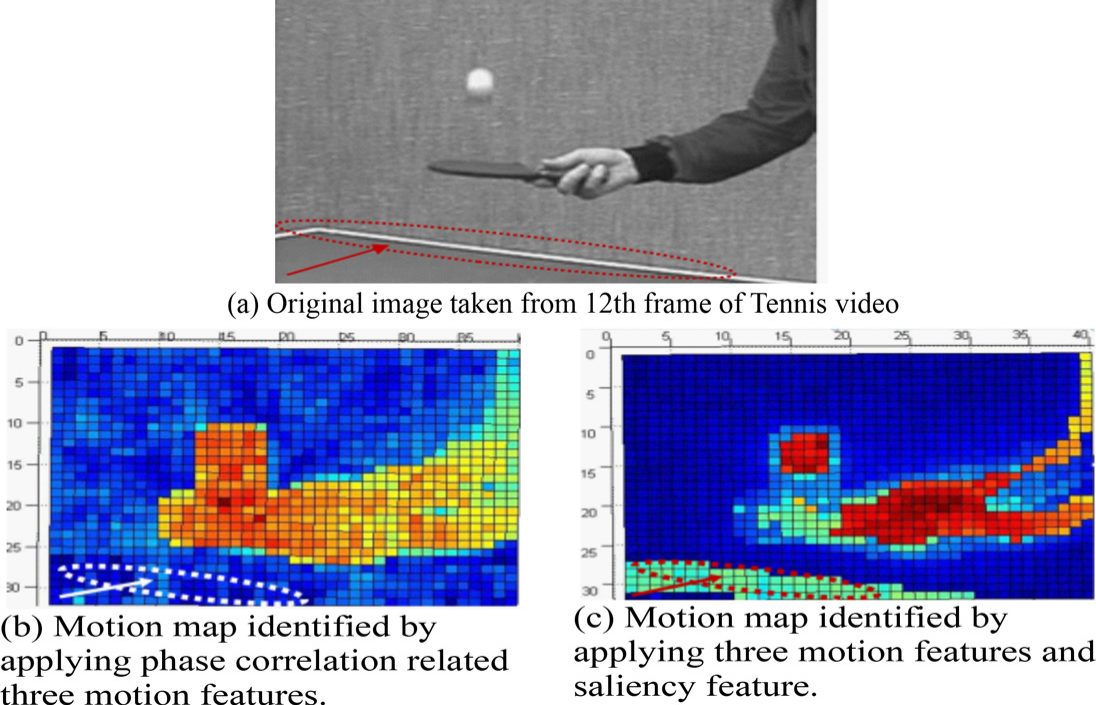
Illustration of motion and salient areas identification with and without saliency feature based cost function.

**Fig 4** illustrates the impact of saliency feature and from **Fig 4(A)**, we clearly depict the ellipse like red marked area as the table tennis court edge (obviously a visually attentive area) that is precisely identified by the saliency feature (red marked ellipse like area in **Fig 4(C)**) although three motion features of phase correlation could not capture the court edge as it does not have any motion (white marked ellipse like area in **Fig 4(B)**). Thus, the proposed combined strategy improves the RD performance by recognizing not only the RDMS- based motion features of phase correlation but also the RDMS- based visually attentive areas in the videos.

### Intermode Selection

**Fig 5:**
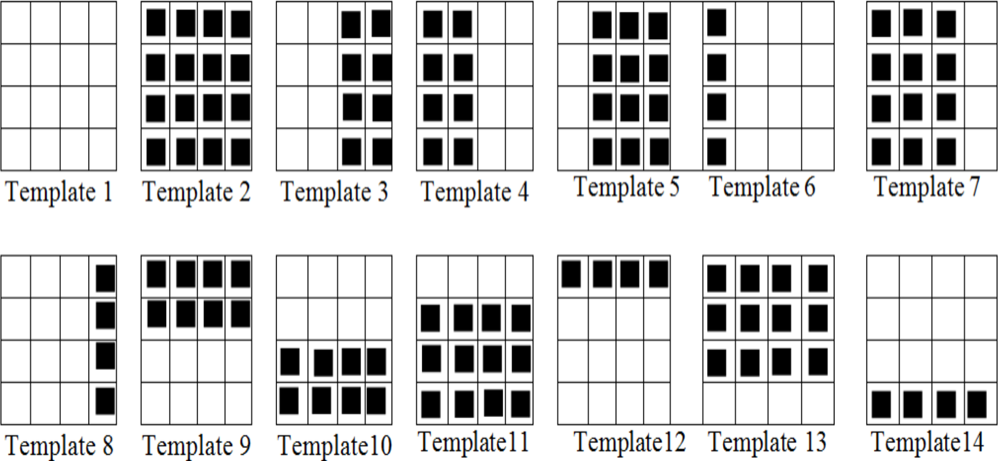
Codebook of the proposed binary pattern templates for subset of inter-mode selection.

In the proposed scheme, we use 64×64 as a CU size. The proposed coding technique selects the best inter-mode at level 0 using LCF without using any phase correlation-based pre-processing. If 32×32 size inter-mode is selected then we activate the proposed RDMS feature to select a subset of inter-modes at level 1 to level 3. For the generation of binary matrix, we exploit each of the 8×8 pixel blocks from the 32×32 block and produce a matrix of 4×4 binary values (*S*(*x*,*y*) in Eq (8)) for each 32×32 (applying threshold). Actually, the matrix of 4×4 binary values is generated using the cost function developed in Eq (7). This cost function generated 4×4 binary matrix is then compared with a codebook of predefined *binary pattern templates* (BPTs) to select a subset of modes (see **Fig 5** and **Table 1**). Each of the templates is constructed with a pattern of RDMS and non- RDMS block (1 and 0 respectively) focusing on the rectangular and regular object shapes at 32×32 block level. In the proposed technique, these adaptively designed binary pattern templates (BPTs) are fully aligned to the HEVC recommended block-partitioning to cover all the modes at 32×32 level. Thus, each individual template selects either a direct mode or a subset of modes which is precisely shown in **Table 1**. In **Fig 5** (for 32×32 level), the cells with black squares present the RDMS (i.e., binary 1) and the rest of the cells present non-RDMS (i.e., binary 0) for mode selection. Similarly in **Table 2** (for 16×16 level), the cells with # symbol present the RDMS and the rest of the cells present non-RDMS for mode selection.

**Table 1:** Mode selection at 32×32 block level based on the codebook of predefined binary pattern templates.

RDMS Based Templates at 32×32 Block Level	Selection of Modes at 32×32 Block Level
**Template 1**	Skip or Inter 32×32
**Template 2**	Intra 16×16 or Inter 16×16
**Template 3 & 4**	Inter {32×16 or 16×16}
**Template 5**	Inter {32×8 or 16×16}
**Template 6**	Inter {32×8}
**Template 7**	Inter {32×24 or 16×16}
**Template 8**	Inter {32×24}
**Template 9 & 10**	Inter {16×32 or 16×16}
**Template 11**	Inter {8×32 or 16×16}
**Template 12**	Inter {8×32}
**Template 13**	Inter {24×32 or 16×16}
**Template 14**	Inter {24×32}

**Table 2:** Subset of mode selection at 16×16 and 8×8 coding depth level based on the patterns of RDMS.

Pattern of RDMS at 16×16 and 8×8 Block Level						Selected Subset of Inter-modes
**#**	**#**	**#**	**#**		**#**	16×8, 8×16 or 8×8
**#**	**#**		**#**	**#**	**#**	
**#**		**#**	**#**			16×8, 8×16 or 8×8
**#**	**#**	**#**				
**#**			**#**	**#**	**#**	16×8 and 8×16
**#**			**#**			
		**#**			**#**	16×8 and 8×16
**#**	**#**					
						16×8 and 8×16
	**#**	**#**				
	**#**	**#**				16×12, 16×4, 12×16 or 4×16
**#**			**#**			

We use a simple similarity metric using the *Hamming Distance* (DH) between the binary matrix of a CU generated by phase correlation and the BPTs in **Fig 5**. We select the best-matched BPT that provides minimum sum of the absolute values of their differences for a CU. The DH, *H*_*k*_ is determined as follows where *S* is the binary motion prediction matrix of a CU comprising 4×4 ‘1’ or ‘0’ combinations and *T*_*k*_ is the *k*-th BPT:

$$H_{k}(x,y)=\sum_{x=0}^{4}\sum_{y=0}^{4}\left|S(x,y)-T_{k}(x,y)\right|$$ (8)

The best-matched *j*-th BPT is selected from all BPTs as follows:

Tj=argmin∀Tk∈BPT(Hk) (9)

The mode selection process from the BPTs at 32×32 and 16×16 coding depth levels is illustrated in **Table 1** and **Table 2** respectively. Once a particular template selects a subset of candidate modes at 32×32 level, only the final mode is decided by their lowest Lagrangian cost function.

Again, at 32×32 level, if any of the 16×16 level mode is selected, we further explore smaller modes including 8×8 at 16×16 coding depth level based on different RDMS block patterns as demonstrated in Table 2 (i.e., presence or absence of binary 1 and 0 in **Table 2**). Then we calculate the minimun value of the Lagrangian cost function (demonstrated in Eq 1) to determine the final mode.

### Threshed Selection

**Fig 6:**
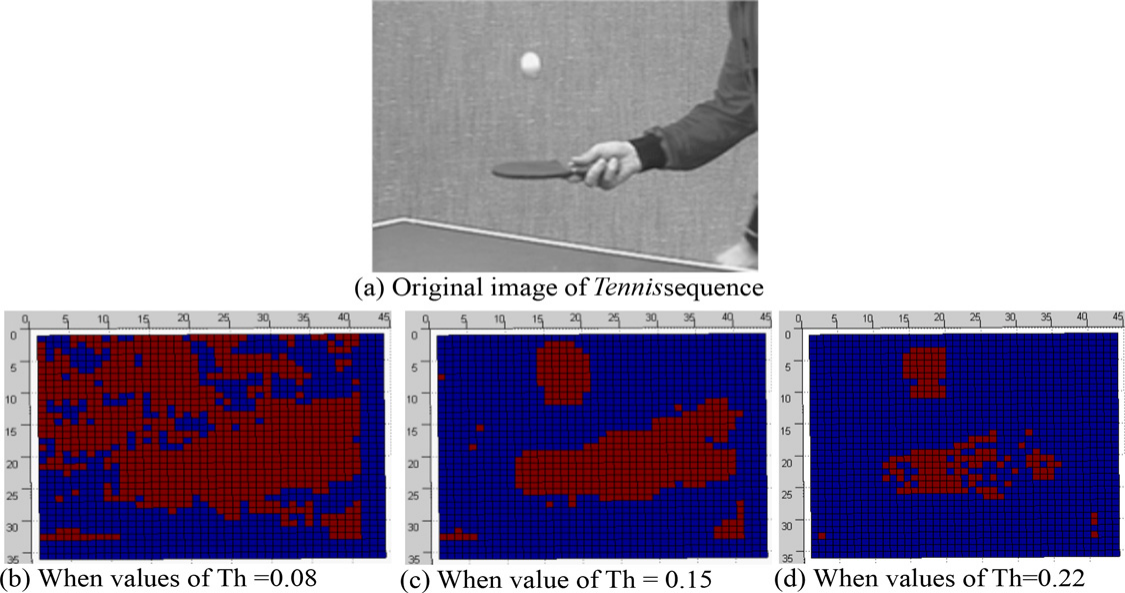
The representation of motion map for various Th values at QP = 28. The reddish blocks in (b~d) indicate the motion regions and the bluish blocks indicate the background regions.

In lieu of selecting thresholds dynamically, we apply the static threshold value and fix it by 0.15 in order to decrease the threshold selection complexity although we consider both the homogeneous and heterogeneous motion regions in the CUs of all sequences. Moreover, the Th (i.e., 0.15) used in the proposed experiment is properly fitted with a variety of test sequences and test conditions. To investigate the implication of the Th used in our algorithm, we apply it for the similar range of QPs recommended by JCT-VC (i.e., 22, 27, 32 and 37) for different sequences and notice that this Th value can appropriately capture the RDMS based regions with accuracy even for a wide range of video contents, different aspects of object motions, camera motion and resolutions. The acquired RDMS based blocks are then encoded with more bits in order to achieve improved quality. We perform experiments using the proposed algorithm for a number of Th values and we present the experimental results for Th = 0.08, Th = 0.15, and Th = 0.22. **Fig 6** demonstrates the patterns of capturing the motion features from the video contents using these Th values. It is obvious from the figure that using Th = 0.15, the proposed algorithm can detect motion in a more realistic way which is the underlying criteria for mode selection. The reddish blocks in **Fig 6B–6D**) present motion (bluish is the background) and **Fig 6 (C)** shows more real presentation of motion than **Fig 6(B) or 6(D)** in terms of capturing the motion from the original image for *Tennis* sequence in **Fig 6(A)**. It is also noticed from the figure that whether Th value is higher or lower than 0.15, motion detection becomes imperfect. Since this trend is observed for almost all sequences, we fix the value of Th by 0.15. Therefore, the use of Th = 0.15 could be validated through the appropriate identification of motion which is properly aligned to the moving regions of the video contents.

Due to the imbalanced distribution of ECR values Paul *et al*. [25] use a range of thresholds for different bit-rates. Those thresholds could not perform well in the proposed method as the distribution of cost function values is more compact. Therefore, in the proposed technique, we use the fixed value of threshold (i.e., 0.15) for a wide range of bit-rates.

## Experimental Results and Analysis

To verify the performance of the proposed algorithm, experimental results are presented with twelve *standard video sequences* (SVS). These include: six *standard definition* (SD- with resolution (352×288)) videos, four *high definition* (HD- with resolution (1920×1088)) videos, and two multiview (MV- with resolution (640×480)) videos. The sequence names and their coresponding BD- PSNR and BD-Bit Rate (compared to HM) results are presented in the following Results and Discussions Sub-section. We also test other video coding experts’ recommended *standard class video sequences* (SCVS) that include Class A, B, C, D, and E. The names and resolutions of these test sequences and their corresponding BD-PSNR and BD-Bit Rate (compared to HM) results are also presented in the Results and Discussions Sub-section. The sequences taken for the experiment are representative in the sense ranging from of low motion activity to the high motion activity, representing a wide range of contents, different aspects of camera motions and resolutions. We compare the proposed method results with HM of HEVC standard as HM outperforms all the existing mode selection techniques stated in the literature.

### Experimental Setup

In this work, the experiments are conducted by a dedicated desktop machine (with Intel core i7 3770 CPU @ 3.4 GHz, 16 GB RAM and 1TB HDD) running 64 bit Windows operating system. The proposed scheme and HEVC with exhaustive mode selection scheme are developed based on the reference software HM (version 12.1[27]). In order to validate the effectiveness of the proposed fast coding scheme, we implement the proposed method into HM12.1 and test it under the common test conditions. RD performance of both schemes are compared considering the maximum CU size of 64×64 by enabling both symmetric and asymmetric partitioning block size of 64×64 to 8×8 depth levels. Each CU is set as a 64×64 pixel block and we only apply RDMS technique when a 32×32 inter-mode is selected. We use 8×8 pixel blocks for binary matrix generation in the 32×32 block (i.e., a 32×32 block is comprised with 4×4 binary values) for a wide range of bit-rates using QPs: 20, 24, 28, 32 and 36 (i.e., similar range of QPs recommended by JCT-VC). All the sequences are encoded with 25 frame rate and search length ±64 (horizontal and vertical). We use IPPP… format with Group of picture (GOP) 32 for both techniques and two reference frames to encode a P-frame for both techniques. The performance is evaluated based on the difference of encoding time (ΔT), the BD-PSNR, and BD-BR. BD-PSNR and BD-BR are calculated according to [33].

### Results and Discussions

**Table 3** shows the performance comparison results of five recent and most relevant fast mode selection algorithms where all the algorithms obtain significant computational time savings compared to different implementations of HEVC by increasing bit-rates and reducing PSNR. The summary of the Table confirm that the proposed technique demonstrates better results compared to the existing state-of-the-art methods in terms of improving rate-distortion performance. Although the time savings for the proposed method is a bit lower than the method used in [9], the bit-rate reduction and PSNR gain are more significant. Compared to the results of other methods mentioned in **Table 3**, the proposed technique shows improved performance in terms of bit-rate reduction. The average PSNR gain for the proposed technique is also higher than all other methods presented in the table (only equal to Shen *et al*.[21]). The overall results of **Table 3** also reveal that the proposed method demonstrates improved performance compared to HM12.1 by saving 34% computational time on average with insignificant bit rate increment and negligible PSNR reduction. The terms BD-BR and BD-PSNR in Table 3 denote BD-Bit Rate and BD-PSNR respectively.

**Table 3:** Performance comparison of different fast mode selection algorithms compared to HEVC encoder in terms of BD-Bit Rate (BD-BR), BD-PSNR and computational time.

Algorithms	BD-BR(%)	BD-PSNR (dB)	Average Time Savings (%)	Remarks
**Correa *et al*.[9], 2011**	5.70	- 0.80	50	03 videos
**Shen *et al*.[21], 2013**	0.60	- 0.01	38	15 videos
**Hou *et al*.[18], 2014**	0.50	- 0.08	30	17 videos
**Pan *et al*. [20], 2014**	0.32	- 0.11	35	19 videos
**Ahn *et al*. [15], 2015**	1.20	-	46	18 videos
**Proposed Method**	0.13	- 0.01	32	12 videos (SVS)
**Proposed Method**	0.10	-0.01	34	14 videos(SCVS)

If any method exhaustively checks all the options in a level to select a particular option, theoretically it should necessitate more computational time. This complexity increases multiple times if any technique has to explore all modes in more depth levels to select a particular mode. Therefore, for all sequences, we compare the average number of modes selected in each CU by HM12.1 and the proposed method. The results in **Table 4** shows that HM checks more options in all cases and normally requires more computational time. From **Table 4**, the overall average percentage of encoding time savings for the SVS by the proposed method is 39.85, while for the SCVS, this percentage is 41.44. The reason behind this acquisition is the efficient subset of intermode selection with simple criteria. However, we cannot ignore the pre-processing stages of the proposed method and by calculation we find that over all sequences on average 6.81% extra encoding time is required to execute phase correlation and saliency related pre-processing overheads (see **Fig 1**). Thus, theoretically we anticipate to acquire 33.04% and 34.63% computational time savings on average for SVS and SCVS respectively.

**Table 4:** A theoretical analysis- percentage of time savings by the proposed method (against HM) for different sequence types based on average number of Inter-modes selected per CU.

Name of the Sequences	Average no. of inter-modes selected per CU by HM	Average no. of inter-modes selected per CU by proposed method	Average Percentage (%) of time saving
*Tennis*	20.15	11.07	45.06
*Paris*	18.05	10.26	43.15
Average time saving for SD type video			44.10
*Pedestrian*	20.68	12.34	40.32
*Parkrun*	19.21	11.39	40.70
Average time saving for HD type video			40.51
*Exit*	20.66	13.16	36.30
*Ballroom*	18.12	12.04	33.55
Average time saving for MV type video			34.92
Average time saving for the standard video sequences (SVS)			39.85
*Class- A*	21.05	12.23	41.90
*Class- B*	18.56	12.18	34.37
*Class-C*	19.78	11.29	42.92
*Class-D*	18.96	10.13	46.57
Average time saving for the standard class video sequences (SCVS)			41.44

**Fig 7:**
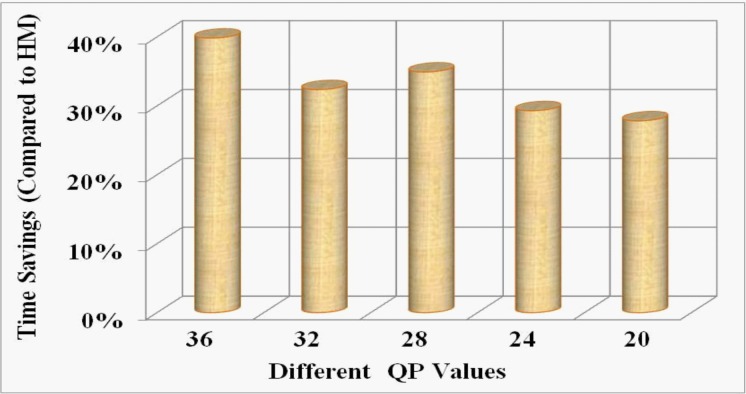
Illustration of overall average time savings by the proposed method against HM at different bit-rates for SVS.

**Fig 8:**
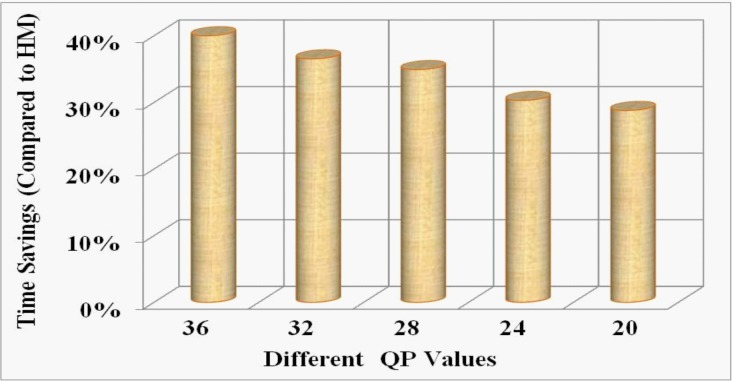
Illustration of overall average time savings by the proposed method against HM at different bit-rates for SCVS.

**Fig 9:**
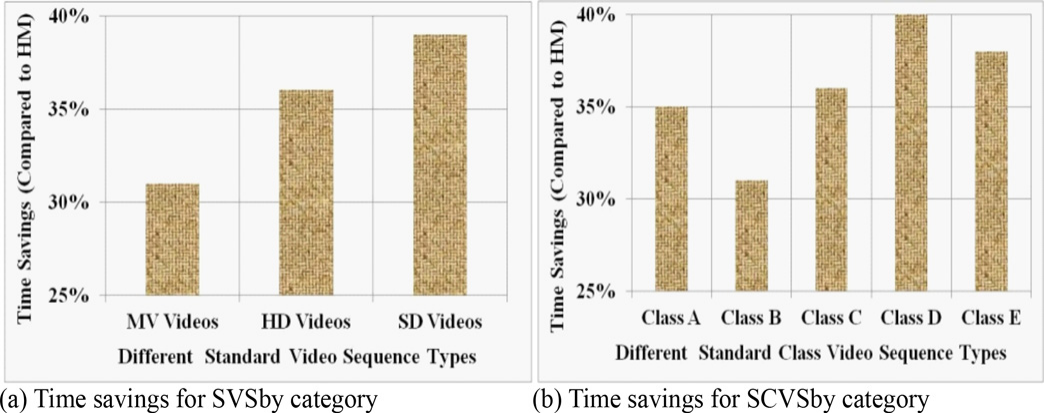
Illustration of overall average time savings by the proposed technique against HM based on different video categories. In (a), the numbers of videos used in HD, MV and SD are 4, 2 and 6 respectively, while in (b), these numbers for Class A, B, C, D and E are 1, 4, 2, 4, and 3 respectively.

The experimental evaluation reveals that over twelve different SVS, and for a wide range of bit-rates, the proposed method reduces on average 32.21% (range: 28%-38%) overall computational time as shown in **Fig 7**. For the SCVS, we experimentally achieve 34.14% (range: 27%-39%) overall encoding time savings on average which is shown in in **Fig 8**. The equation for the time savings (Δ*T*) is defined as:

$$\Delta T=\frac{{(T}_{0}-T_{p})}{T_{0}}\times 100\%$$ (10)

where *T*_*0*_ and *T*_*p*_ denote the total encoding time consumed by HM and the proposed method respectively. For comprehensive performance test, we execute the computational time analysis of both techniques based on video categories for SVS, and find that the proposed method achieves on average 34% encoding time savings compared to HM12.1 as shown in **Fig 9(A)**. For the SCVS, this percentage is 35 which is shown in **Fig 9(B)**. The figure also reveals that the proposed technique saves more time for SD type and Class D type videos compared to other types while using SVS and SCVS respectively.

**Fig 10:**
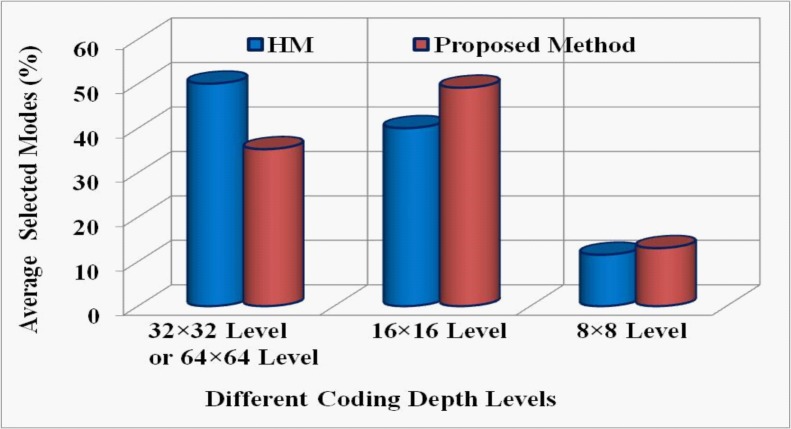
HM and proposed method based average mode selection at three coding depth levels.

**Fig 11:**
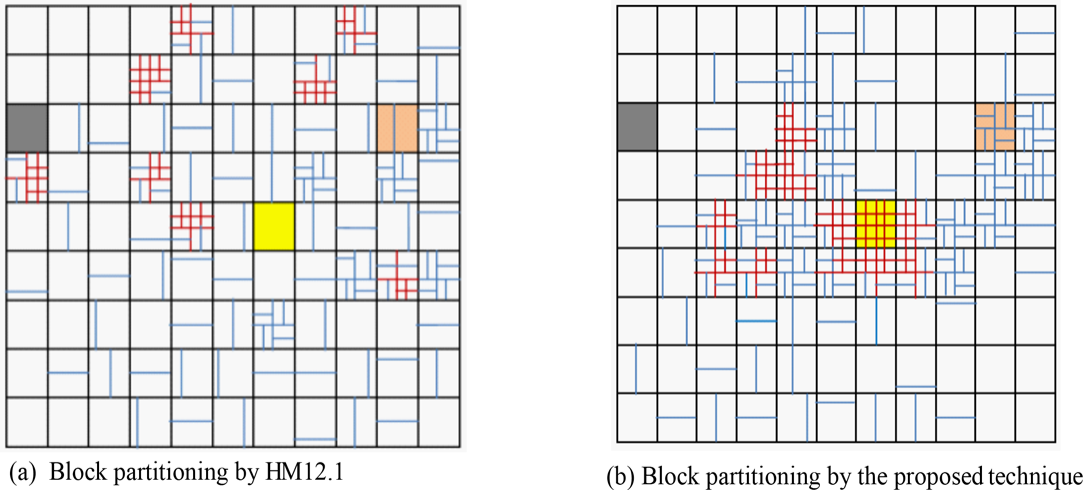
Block partitioning for the 12^th^ frame of the *Tennis* video at QP = 24 with HM and the Proposed method.

**Fig 10** shows the average percentage of three different depth level modes for all sequences at QP = 20 to 36. For the proposed method, the higher depth level modes (16×16 and 8×8) are exploited for the RDMS based CUs due to acquire similar/improved RD performance. **Fig 11** reveals the block partitioning example for the 12^th^ frame of *Tennis* video at QP = 24 by both schemes. If we compare **Fig 11(A) and 11(B)** we observe an identical block partitioning framework for the block at (3, 1) position (dark block) and (3, 10) position (pink block) in both methods. Both techniques consider these blocks with no motion and simple motion respectively (justified by **Fig 2**) and thus, partition accordingly. However, the partitioning evidence of yellow marked CUs are distinguishable from each other.

First, we consider the block at (5, 7) position in **Fig 2** and observe the complex motion therein. The HM could not capture any motion in that block and keeps it un-partitioned, while, the proposed technique considers it as a complex motion block (**Fig 2 (D))** with the the highest ECR and saliency values (**Fig 2(E) and 2(F)**). Therefore the proposed technique partitions that block with higher level modes. This approach of RDMS based mode selection reflects on achieving the similar RD performance for a wide range of bit-rates. In **Fig 11**, the blocks with red partitions indicate that at least one 8×8 level mode is selected by the proposed method for partitioning that block.

**Fig 12:**
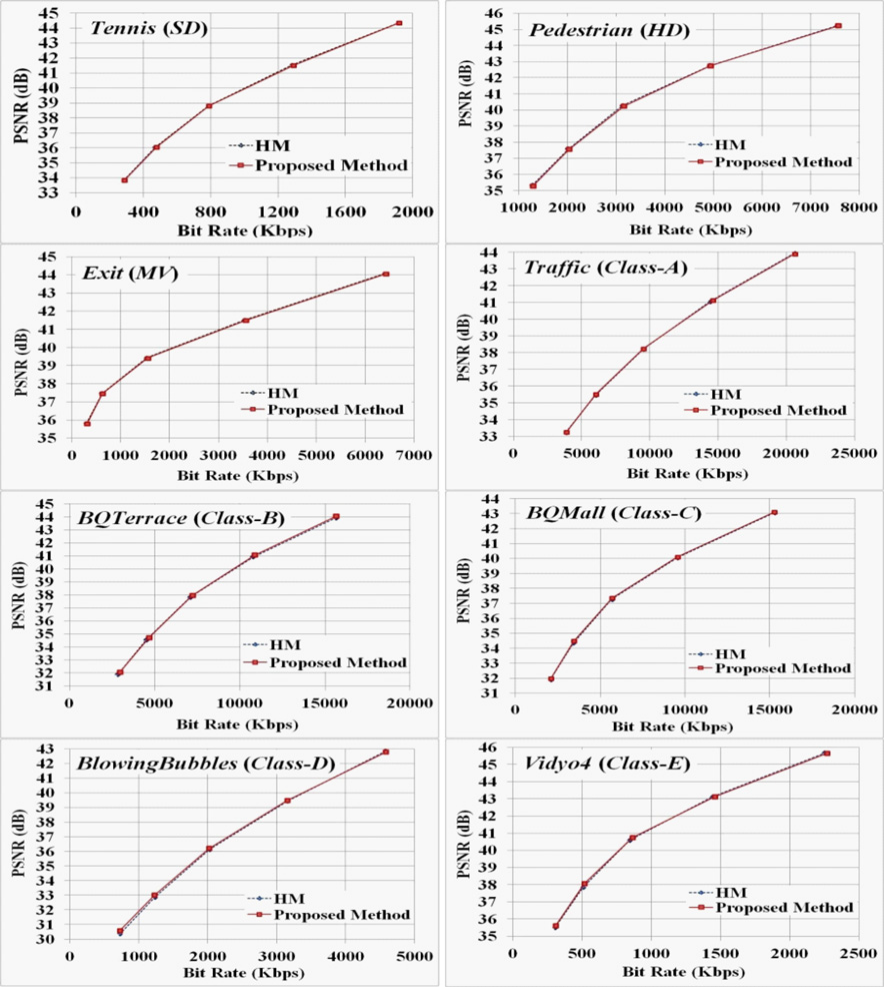
Comparative study on RD performance by HM12.1 and the proposed method for a wide range of bit-rates.

To test the performance of the proposed method objectively using SVS, we first compare the RD performances against HM using three sequences (one SD, HD and MV category) for a wide range of bit-rates as demonstrated in **Fig 12**. For these sequences, the proposed method shows similar RD performance with HM especially caring about the RDMS based blocks and partitioning those blocks with appropriate coding depth level modes.

From the experimental outcomes of **Fig 12**, we observe the similar RD performance for five SCVS (*Traffic*, *BQTerrace*, *BQMall*, *BlowingBubbles*, and *Vidyo4*- i.e., one from each Class type). **Table 5** and **Table 6** present the overall performance comparison results of the proposed technique for twelve different SVS and fourteen different SCVS respectively. The results reveal that compared to the mode selection approach in HM12.1, the proposed technique achieves an almost similar RD performance (small average reduction of 0.01dB PSNR for SVS and SCVS) with a negligible bit-rate increment of 0.13% and 0.10% for SVS and SCVS respectively.

**Table 5:** Performance comparison of proposed technique compared to HM12.1 using BD-BR and BD-PSNR for the SVS.

Sequence Resolutions	Name of the Sequences	BD-PSNR(dB)	BD-BR(%)
**352×288**	*Tennis*	-0.01	0.08
**352×288**	*Silent*	-0.01	0.14
**352×288**	*Waterfall*	-0.01	0.12
**352×288**	*Tempete*	-0.01	0.11
**352×288**	*Paris*	-0.01	0.10
**352×288**	*Bridgeclose*	-0.01	0.14
*Average*		-0.01	0.11
**1920×1088**	*Pedestrian*	-0.01	0.15
**1920×1088**	*Bluesky*	-0.02	0.21
**1920×1088**	*Parkrun*	-0.02	0.16
**1920×1088**	*Rushhour*	-0.01	0.14
*Average*		-0.02	0.16
**640×480**	*Ballroom*	-0.01	0.13
**640×480**	*Exit*	-0.01	0.11
***Average***		-0.01	0.01
**Overall Average**		-0.01	0.13

**Table 6:** Performance comparison of proposed technique compared to HM12.1 using BD-BR and BD-PSNR for the SCVS.

Class	Sequence Resolutions	Name of the Sequences	BD-PSNR(dB)	BD-BR(%)
**A**	2560×1600	*Traffic*	-0.01	0.14
**B**	1920×1080	*BasketballDrive*	-0.01	0.11
**B**	1920×1080	*BQTerrace*	0.00	0.04
**B**	1920×1080	*Cactus*	-0.02	0.18
**B**	1920×1080	*Tennis*	0.00	0.07
**C**	832×480	*BasketballDrill*	-0.01	0.10
**C**	832×480	*BQMall*	0.00	0.01
**D**	416×240	BasketballPass	-0.02	0.17
**D**	416×240	BlowingBubbles	-0.01	0.09
**D**	416×240	BQSquare	-0.01	0.13
**D**	416×240	Flowervase	0.00	0.07
**E**	1280×720	Vidyo1	-0.01	0.10
**E**	1280×720	Vidyo3	-0.01	0.15
**E**	1280×720	Vidyo4	-0.01	0.09
**Overall Average**			-0.01	0.10

### Subjective Quality Assessment

It is widely accepted that objective assessment based on PSNR does not always provide reliable assessments of video quality, since a higher PSNR may not always promise better video quality [34]. Therefore, it has become common practice in international coding-standard activities to combine both objective and subjective assessments in evaluating and comparing video coding algorithms. To compare the perceptual performance, we perform a subjective test using *Double-Stimulus Continuous Quality Scale* (DSCQS) assessment using the test conditions of [35]. We used a number of video sequences serially to accomplish this test. The viewers are asked to rate the quality of two video sequences known as “H” and “P” on a continuous scale ranging between “Excellent” and “Bad”. The options “H” and “P” are the reconstructed video sequences using HM12.1 and the proposed algorithm. The experimental results reveal that viewers recognize and mark them as very similar.

**Fig 13:**
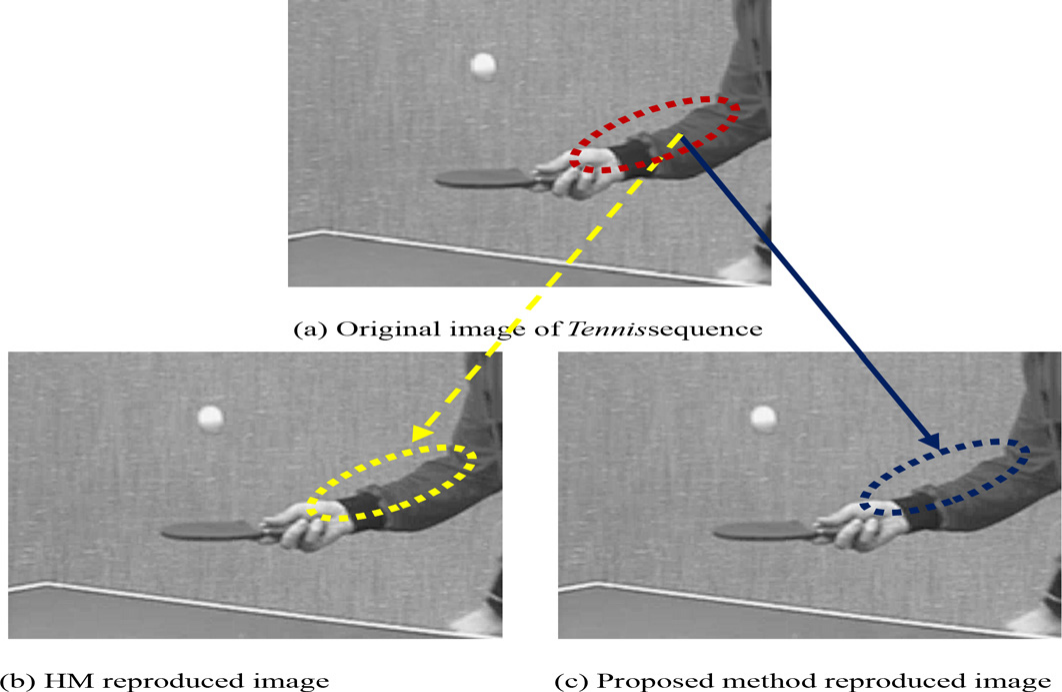
Subjective quality evaluation for HM12.1 and the proposed technique for *Tennis* video sequence. The figures are achieved from the 20^th^ frame of the *Tennis* video at the same bit-rate.

As an example, **Fig 13(A)** shows the original image of *Tennis* video taken for subjective quality test and **Fig 13(B) and 13(C)** illustrate the reproduced images by HM and the proposed method respectively. To present the comparison in image quality let us concentrate on the cuff and sleeve sections of the shirt in the three images which are marked by the red, yellow, and blue ellipses respectively. It can be perceived that the three ellipse marked sections have almost similar image quality. The figures (**Fig 13(B) and 13(C)**) are achieved from the 20^th^ frame of the *Tennis* video at the same QP (i.e., 24) and for both techniques, we notice the same bits per frame (52508) and PSNR (41.52) values. It was presented earlier in this manuscript that the proposed method requires less encoding time. Hence it can be concluded that the proposed technique shows significant computational time savings compared to HM12.1 with similar image quality for a wide range of bit- rates. Due to this phenomenon, the proposed implementation is expected to become more suitable for all real time video coding applications especially for a number of electronic devices with limited processing power and battery capacity.

## Conclusion

Due to high computational complexity, HEVC could not facilitate numerous electronic devices with limited processing and battery capacity. To address this limitation, this work presents a novel video coding framework for the performance improvement of HEVC encoder. The proposed fast mode selection scheme exploits RDMS based mode selection technique comprising with phase correlation based three different motion features and human visual attention based saliency feature. The motion features focus on three different aspects of motions and the saliency feature captures attentive regions that are sensitive to the human visual syatem for quality assessment. We mathematically drive an adaptive weighted cost function by innovatively combining features in order to determine a subset of inter-modes using predefined RDMS based binary pattern templates. The Lagrangian optimization criterion is applied only on the selected subset for the final mode decision. Compared to the mode selection approach in HM12.1, the proposed technique demonstrates similar RD perfoemance (very small reduction of 0.01dB PSNR or, 0.10% bit-rate increment) for a wide range of bit-rates. It could also reveal 34% overall average computational time reduction (ranging 27%-39% compared to HM) which is expected to become more suitable for many real time video coding applications.
